# Cross-Sectional Analysis of the 1039 U.S. Physicians Reported to the National Practitioner Data Bank for Sexual Misconduct, 2003–2013

**DOI:** 10.1371/journal.pone.0147800

**Published:** 2016-02-03

**Authors:** Azza AbuDagga, Sidney M. Wolfe, Michael Carome, Robert E. Oshel

**Affiliations:** 1 Health Research Group, Public Citizen, Washington, District of Columbia, United States of America; 2 National Practitioner Data Bank (Retired), Department of Health and Human Services, Silver Spring, Maryland, United States of America; University of California San Diego, UNITED STATES

## Abstract

**Background:**

Little information exists on U.S. physicians who have been disciplined with licensure or restriction-of-clinical-privileges actions or have had malpractice payments because of sexual misconduct. Our objectives were to: (1) determine the number of these physicians and compare their age groups’ distribution with that of the general U.S. physician population; (2) compare the type of disciplinary actions taken against these physicians with actions taken against physicians disciplined for other offenses; (3) compare the characteristics and type of injury among victims of these physicians with those of victims in reports for physicians with other offenses in malpractice-payment reports; and (4) determine the percentages of physicians with clinical-privileges or malpractice-payment reports due to sexual misconduct who were not disciplined by medical boards.

**Methods and Results:**

We conducted a cross-sectional analysis of physician reports submitted to the National Practitioner Data Bank (NPDB) from January 1, 2003, through September 30, 2013. A total of 1039 physicians had ≥ 1 sexual-misconduct–related reports. The majority (75.6%) had only licensure reports, and 90.1% were 40 or older. For victims in malpractice-payment reports, 87.4% were female, and “emotional injury only” was the predominant type of injury. We found a higher percentage of serious licensure actions and clinical-privileges revocations in sexual-misconduct–related reports than in reports for other offenses (89.0% vs 68.1%, *P* = < .001, and 29.3% vs 18.8%, *P* = .002, respectively). Seventy percent of the physicians with a clinical-privileges or malpractice-payment report due to sexual misconduct were not disciplined by medical boards for this problem.

**Conclusions:**

A small number of physicians were reported to the NPDB because of sexual misconduct. It is concerning that a majority of the physicians with a clinical-privileges action or malpractice-payment report due to sexual misconduct were not disciplined by medical boards for this unethical behavior.

## Introduction

Physician sexual misconduct dangerously exploits the physician-patient relationship. This behavior “may be verbal or physical, and may include expressions of thoughts and feelings or gestures that are sexual or that reasonably may be construed by a patient or patient’s surrogate as sexual” [1]. The long-standing prohibition against sexual relations with patients goes back to the Hippocratic oath in the fourth century BC: “I will come for the benefit of the sick, remaining free of … sexual relationships with both female and male persons” [2]. The Council on Ethical and Judicial Affairs for the American Medical Association determined it is unethical for a physician to have a romantic relationship or sexual contact with a current patient and that a sexual relationship with a former patient is also unethical if the physician “uses or exploits trust, knowledge, emotions, or influence” derived from the prior physician-patient relationship [3].

Although this prohibition on physician-patient sexual relations is based on evidence that such relations harm patients and endanger their medical care [4–9], physician sexual misconduct has received inadequate attention from researchers [10]. Two recent studies [11,12] examined, in part, physician sexual misconduct among Canadian physicians, but no such U.S. national-level analyses have been published on this important public health problem since Public Citizen’s 1998 study, limited to medical board licensure actions [13].

To the best of our knowledge, this study is the first to examine physician sexual misconduct in the U.S. using the National Practitioner Data Bank (NPDB)—the only national repository of legally required reports subject to accuracy review concerning disciplinary actions by state medical boards or clinical peer review committees, or malpractice payments resulting from physician wrongdoing [14]. Although the Federation of State Medical Boards compiles information on physician licensure disciplinary actions [15], only the NPDB collects comprehensive information on peer reviewed clinical-privileges disciplinary actions and malpractice payments in addition to licensure actions [16]. Therefore, the NPDB provides the only comprehensive and definitive data available on sexual misconduct in the context of disciplined physicians in the U.S.

This study aimed to: (1) determine the number of these physicians and compare their age groups’ distribution with that of the general U.S. physician population; (2) compare the type of disciplinary actions taken against these physicians with actions taken against physicians disciplined for other offenses; (3) compare the characteristics and type of injury among victims of these physicians with those of victims in reports for physicians with other offenses in malpractice-payment reports; and (4) determine the percentages of physicians with clinical-privileges or malpractice-payment reports due to sexual misconduct who were not disciplined by medical boards.

## Material and Methods

### Design and Data Sources

This study was a retrospective cross-sectional analysis of de-identified physician data from the NPDB’s public-use file, published by the U.S. Department of Health and Human Services [17]. A national database established under the Health Care Quality Improvement Act of 1986 [18], the NPDB includes reports of (a) all adverse licensure actions taken against physicians by state medical boards (referred to hereafter as “licensure reports”); (b) adverse restriction-of-clinical-privileges actions lasting more than 30 days taken against physicians by clinical peer review committees in hospitals, nursing homes, etc., including similar panel membership actions taken by managed care organizations (referred to hereafter as “clinical-privileges reports”); and (c) all malpractice payments made on behalf of physicians (referred to hereafter as “malpractice-payment reports”) [16]. Notably, the three report types have some variables in common (such as physician age groups), but differ with respect to the other variables as described in later parts of our methods section.

Although most of our analyses were at the report level, we conducted physician-level analyses for the number of sexual-misconduct–related report types and physician age groups. Comparisons of sexual-misconduct–related reports with reports related to other offenses were conducted for the type of disciplinary action for licensure and clinical-privileges reports, as well as for victim characteristics and the type of victim injury for malpractice-payment reports only.

We also compared physicians with sexual-misconduct–related reports with the general U.S. physician population (using data from the Federation of State Medical Boards) [19] with respect to age groups (Supplemental Methods in S1 File).

### Physician Report Selection Criteria

As noted earlier, the NPDB receives reports of disciplinary actions taken by state medical boards or clinical peer review committees and malpractice payments made on behalf of physicians by malpractice insurance companies or others. The NPDB itself takes no action and makes no findings. It simply receives reports and disseminates them to those authorized to receive the information. The NPDB has made explicit sexual-misconduct codes available for use in licensure and clinical-privileges reports since September 2002 and in malpractice-payment reports since January 2004 [20]. When our study was initiated, the NPDB file included data through September 30, 2013. Therefore, our study period for licensure and clinical-privileges reports was from January 1, 2003, and for malpractice-payment reports from January 1, 2004, through September 30, 2013.

We identified all sexual-misconduct–related licensure, clinical-privileges, and malpractice-payment reports (Supplemental Methods in S1 File) for all physicians, including M.D., D.O., and intern/resident physicians during the study period. We also identified all reports for other physicians for whom the reported basis for disciplinary action involved offenses other than sexual misconduct during the study period (note: we excluded reports in which no basis for action was reported). We used these other-offenses–related physician reports to perform comparisons with physician sexual-misconduct–related reports.

### Physician and Victim Characteristics

All three report types included physician age group. For sexual-misconduct physician-level analyses we used age group as reported in the earliest sexual-misconduct–related report during the study period. We categorized age groups as under 40, 40 to 49, 50 to 59, 60 or older, and unknown. Only malpractice-payment reports had information about victim age group (which we categorized as 1 to 20, 20 to 39, 40 to 59, or 60 to 79 years, or unknown), sex, and setting (categorized as outpatient, inpatient, both inpatient and outpatient, or unknown).

### Physician Licensure Disciplinary Actions

For licensure reports, we examined the reported types of disciplinary action taken by the medical boards. Because each licensure report may list up to five licensure disciplinary actions, we calculated the number of reports that included each of these actions, regardless of the order in which they were listed. We further reported the number of reports that included serious versus nonserious licensure actions. We defined a serious licensure action based on the presence of any of the following 11 licensure actions: “revocation of license,” “probation of license,” “suspension of license,” “summary/emergency limitation/restriction on license,” “summary/emergency suspension of license,” “voluntary surrender of license,” “limitation or restriction on license/practice,” “denial of license (renewal only),” “voluntary agreement to refrain or suspend pending completion of investigation,” “denial of initial license,” and “voluntary limitation/restriction of license.” We defined a nonserious licensure action based on the absence of a serious action in the report and the presence of at least one of the following four actions: *“*reprimand or censure of license,” “publicly available fine/money penalty (licensure),” “publicly available negative action/finding,” and “other licensure action (not classified).”

### Physician Clinical-Privileges Disciplinary Actions

For clinical-privileges reports, we examined the reported types of disciplinary action taken by clinical peer review committees. Similar to licensure reports, clinical-privileges reports may list up to five adverse actions. Nine different types of adverse actions were found in the reports analyzed in our study: “revocation of clinical privileges/panel membership,” “professional review employment or panel membership firing,” “voluntary surrender of clinical privileges/panel membership under investigation,” “involuntary resignation/panel membership,” “denial of clinical privileges,” “suspension of clinical privileges/panel membership,” “summary/emergency suspension of clinical privileges/panel membership,” “limitation/restriction of procedures/practice area” and “other restriction/limitation of clinical privileges.” We calculated the number of reports that included each of these clinical-privileges actions, regardless of the order in which they were listed in each report. We considered all reported clinical-privileges actions to be serious because nonserious clinical-privileges actions—those limiting the ability of a physician to practice at the reporting entity to 30 days or less—are not reported to the NPDB.

### Type of Victim Injuries in Malpractice-Payment Reports

For malpractice-payment reports, we examined the reported type of injury suffered by the patient. This variable was available only in malpractice-payment reports. Each of these reports listed only one of the following possible 10 types of injuries: “emotional injury only;” “death;” “significant permanent injury;” “major permanent injury;” “major temporary injury;” “quadriplegic, brain damage, lifelong care;” “insignificant injury;” “minor temporary injury;” “minor permanent injury;” or “cannot be determined.”

### Statistical Analysis

Descriptive analyses included frequencies and percentages for study variables. Two-sample two-tailed z-tests were conducted to test differences in age groups’ distributions between physicians with sexual-misconduct reports and the general U.S. physician population. Chi-square or Fisher exact tests were used to test differences in the disciplinary actions against physicians, victim characteristics, and type of victim injury between sexual-misconduct–related reports and physician reports related to other offenses. A significance level of .05 was used for all comparisons. Data were analyzed using SAS version 9.3 (SAS Institute, Inc, Cary, NC).

## Results

Of 100 165 unique physicians with licensure, clinical-privileges, or malpractice-payment reports in the NPDB during the study period, 1039 had one or more sexual-misconduct–related reports (accounting for approximately 1% of the total physicians with one or more of any NPDB reports). Most of these 1039 physicians had only sexual-misconduct–related licensure reports (75.6%) (Table 1). Physicians with only clinical-privileges or malpractice-payment reports accounted for 8.9% and 7.9% of the 1039 physicians, respectively. The remaining 79 (7.6%) physicians had sexual-misconduct reports in two or more of these report types. Moreover, 12.9% of the physicians with sexual-misconduct–related reports had multiple reports of the same type, with licensure reports being the most common report type among these physicians (see Table 1 for more details).

**Table 1:** Physicians With Sexual-Misconduct–Related NPDB Reports, 2003–2013 (Physician-Level Analysis).

Physician with Sexual-Misconduct Reports	No. (%)
**Physicians with ≥ 1 sexual-misconduct**–**related reports of any type**	1039 (100.0)
**Physicians with only one type of sexual-misconduct**–**related reports**	960 (92.4)
≥ 1 licensure reports only	786 (75.6)
≥ 1 clinical-privileges reports only	92 (8.9)
≥ 1 malpractice-payment reports only	82 (7.9)
**Physicians with two or more types of sexual-misconduct**–**related reports**	79 (7.6)
≥ 1 licensure and ≥ 1 clinical-privileges reports only	33 (3.2)
≥ 1 licensure and ≥ 1 malpractice-payment reports only	40 (3.9)
≥ 1 clinical-privileges and ≥ 1 malpractice-payment reports only	3 (.3)
≥ 1 licensure, ≥ 1 clinical-privileges, and ≥ 1 malpractice-payment reports	3 (.3)
**Physicians with ≥ 2 sexual-misconduct**–**related reports of the same type**	134 (12.9)
≥ 2 licensure reports	100 (9.6)
≥ 2 clinical-privileges reports	8 (.8)
≥ 2 malpractice-payment reports	26 (2.5)
**Physicians with ≥ 1 sexual-misconduct**–**related clinical-privileges and/or malpractice-payment reports**	253 (24.4)
Physicians with ≥ 1 sexual-misconduct–related clinical-privileges or malpractice-payment reports, but no sexual-misconduct–related licensure reports^a^	177 (70.0)
**Physicians with ≥ 1 sexual-misconduct**–**related licensure reports**	862 (83.0)
**Physicians with ≥ 1 sexual**-**misconduct**–**related clinical-privileges reports**	131 (12.6)
**Physicians with ≥ 1 sexual**-**misconduct**–**related malpractice-payment reports**	128 (12.3)

^a^ Percentage for this count is based on the counts in the preceding row.

### Physician Characteristics

There were statistically significant differences in the proportions of most age groups for physicians with sexual-misconduct–related reports compared with those for the general U.S. physician population: 9.6% of physicians with sexual-misconduct–related reports vs 23.3% for the U.S. physician population were aged 20 to 39 (*P* < .001); 28.6% vs 24.9% were aged 40 to 49 (*P* = .006); and 35.2% vs 24.8% were aged 50 to 59 (*P* < .001) (Table 2). Thus, there were significantly more physicians aged 40 to 49 and 50 to 59 with sexual-misconduct–related reports and fewer 20- to 39-year-old physicians with sexual-misconduct–related reports than represented by those age groups in the general physician population.

**Table 2:** Comparison of Age Group Distribution of Physicians With Sexual-Misconduct–Related Reports during the Study Period With the U.S. General Physician Population (Physician-Level Analysis).

Physician Characteristics	Physicians With Sexual-Misconduct–Related Reports No. (%)^a^	General U.S. Physician Population No. (%)^a^	Expected Physicians With Sexual-Misconduct–Related Reports^b^ No.	*P* Value
**Age Group**				
All	1039 (100.0)	850 085^d^ (100.0)		
20–39	100 (9.6)	198 174 (23.3)	242	< .001
40–49	297 (28.6)	211 668 (24.9)	259	.006
50–59	366 (35.2)	210 797 (24.8)	258	< .001
60 or older	273 (26.3)	207 515 (24.4)	254	.15
Unknown	3 (.3)	21 931 (2.6)	27	< .001

^a^ Percentages may not add up to 100 due to rounding.

^b^ Expected counts assume the same age percentage distribution for physicians with sexual-misconduct–related reports as in the general U.S. physician population.

### Victim Characteristics in Malpractice-Payment Reports

For malpractice-payment reports, there were 167 sexual-misconduct–related reports submitted for 128 physicians, accounting for a tiny proportion (.2%) of the total 110 738 physician reports during the study period. As shown in Table 3, the proportion of victims between 20 and 39 years of age in sexual-misconduct–related reports was double that for the same-aged victims in malpractice-payment reports related to other offenses (46.1% vs 22.6%, *P* < .001). Conversely, the proportion of victims 60 and older was approximately one-seventh as high in sexual-misconduct–related reports as in reports related to other offenses (3.0% vs 21.9%, *P* < .001). The proportions of victims under 20 and those between 40 and 59 years of age for sexual-misconduct–related reports and reports related to other offenses were not significantly different. The proportion of females listed as victims in sexual-misconduct–related reports was significantly greater than that in malpractice-payment reports related to other offenses (87.4% vs 54.6%, *P* < .001).

**Table 3:** Victim Characteristics and Type of Victim Injury in Physician Sexual-Misconduct–Related vs Physician Malpractice-Payment Reports Related to Other Offenses (Report-Level Analysis).

Victim Characteristics	Sexual-Misconduct–Related Reports (n = 167)^a^	Other-Offenses–Related Reports (n = 110 571)^b^	
	No. (%)^c^	No. (%)^c^	*P* Value
**Age Group**			
1–19 years	15 (9.0)	15 799 (14.3)	.08
20–39 years	77 (46.1)	24 940 (22.6)	< .001
40–59 years	53 (31.7)	39 097 (35.4)	.58
60–79 years	5 (3.0)	24 180 (21.9)	< .001
Unknown	17 (10.2)	6555 (5.9)	.01
**Sex**			
Female	146 (87.4)	60 311 (54.6)	< .001
Male	21 (12.6)	48 130 (43.5)	< .001
Unknown	0 (.0)	2130 (1.9)	.08
**Setting**			
Inpatient	14 (8.4)	49 680 (44.9)	< .001
Outpatient	140 (83.8)	43 725 (39.5)	< .001
Both inpatient and outpatient	5 (3.0)	10 028 (9.1)	.01
Unknown	8 (4.8)	7138 (6.5)	.38
**Type of Injury**^d^			
Emotional injury only	137 (82.0)	1632 (1.5)	< .001
Death	2 (1.2)	35 340 (32.0)	< .001
Significant permanent injury	1 (.6)	16 771 (15.2)	< .001
Major permanent injury	1 (.6)	11 758 (10.6)	< .001
Major temporary injury	4 (2.4)	11 009 (10.0)	.001
Quadriplegic, brain damage, lifelong care	0 (.0)	5629 (5.1)	.003
Insignificant injury	4 (2.4)	1836 (1.7)	.37
Minor temporary injury	11 (6.6)	10 444 (9.5)	.19
Minor permanent injury	3 (1.8)	13 232 (12.0)	< .001
Cannot be determined from available report	4 (2.4)	2920 (2.6)	>.99

^a^ Reports are for 128 unique physicians with sexual-misconduct–related malpractice-payment reports.

^b^ Reports are for 80 741 unique physicians with malpractice-payment reports related to other offenses.

^c^ Percentages may not add up to 100 due to rounding.

^d^ Only one type of injury code is permitted in malpractice-payment reports.

The majority of sexual-misconduct–related malpractice-payment reports concerned incidents in the outpatient setting, and this proportion was significantly greater than that for malpractice-payment reports related to other offenses (83.8% vs 39.5%, *P* < .001). Conversely, far fewer sexual-misconduct–related malpractice-payment reports concerned incidents in the inpatient setting compared with malpractice-payment reports related to other offenses (8.4% vs 44.9%, *P* < .001). Smaller proportions of sexual-misconduct reports concerned both inpatient and outpatient setting in both report types (3.0% and 9.1% in sexual-misconduct and malpractice-payment reports related to other offenses, respectively, *P* = .01).

### Reported Physician Licensure Disciplinary Actions

Only 974 (2.9%) of the total 33 637 physician licensure reports during the study period were sexual-misconduct–related. Overall, serious licensure actions made up a greater proportion of the disciplinary actions taken in sexual-misconduct–related licensure reports compared with licensure reports related to other offenses (89.0% vs 68.1%, *P* < .001) (Table 4). Of all major types of serious licensure actions, license suspension was noted in 22.9% of sexual-misconduct–related reports compared with 16.0% of reports related to other offenses (P < .001). License revocation was the second-most-frequent serious licensure action in sexual-misconduct–related reports, representing a significantly greater proportion compared with reports related to other offenses (16.2% vs 7.6%, *P* < .001). Additionally, summary/emergency license suspension and license/practice limitation or restriction were noted in higher proportions of sexual-misconduct–related reports compared with reports related to other offenses (13.7% vs 4.9%, *P* < .001; and 10.5% vs 7.6%, *P* = .001, respectively). Conversely, nonserious licensure actions of reprimand or censure and of publicly available negative action or finding were noted in lower proportions of sexual-misconduct–related reports compared with physician reports related to other offenses (13.9% vs 26.3%, *P* < .001; and .2% vs 1.1%, *P* = .01, respectively).

**Table 4:** Licensure Disciplinary Actions Taken Against Physicians in Sexual-Misconduct–Related vs Other-Offenses–Related Physician Licensure Reports (Report-Level Analysis).

Licensure Actions^a^	Sexual-Misconduct–Related Reports (n = 974)^b^	Other- Offenses–Related Reports (n = 32 663)^c^	
	No. (%)	No. (%)	*P* Value
**Reports with one or more serious licensure disciplinary actions**	867 (89.0)	22 256 (68.1)	< .001
**Reports with no serious licensure disciplinary actions**	107 (11.0)	10 407 (31.9)	< .001
**Specific types of serious licensure disciplinary actions**			
Reports with revocation of license action	158 (16.2)	2497 (7.6)	< .001
Reports with probation of license action	162 (16.6)	6257 (19.2)	.05
Reports with suspension of license action	223 (22.9)	5218 (16.0)	< .001
Reports with summary/emergency limitation/restriction on license action	5 (.5)	75 (.2)	.07
Reports with summary/emergency suspension of license action	133 (13.7)	1612 (4.9)	< .001
Reports with voluntary surrender of license action	120 (12.3)	3705 (11.3)	.34
Reports with limitation or restriction on license/practice action	102 (10.5)	2506 (7.6)	.001
Reports with voluntary agreement by physician to refrain from practicing/suspension of license PCI action	4 (.4)	94 (.3)	.48
Reports with denial of license (renewal only) action	5 (.5)	772 (2.4)	.001
Reports with denial of initial license action	9 (.9)	533 (1.6)	.08
Reports with voluntary limitation/restriction of license action	16 (1.6)	334 (1.0)	.06
**Specific types of nonserious licensure disciplinary actions**			
Reports with reprimand or censure license action	135 (13.9)	8603 (26.3)	< .001
Reports with publicly available fine/money penalty licensure action	78 (8.0)	3085 (9.4)	.13
Reports with publicly available negative action/finding	2 (.2)	346 (1.1)	.01
Reports with other licensure (not classified) action	73 (7.5)	4051 (12.4)	< .001

Abbreviation: PCI, pending completion of an investigation.

^a^ Each report can have up to five actions.

^b^ Reports are for 862 unique physicians with sexual-misconduct–related licensure reports.

^c^ Reports are for 22 569 unique physicians with other-offenses–related licensure reports.

### Reported Physician Clinical-Privileges Disciplinary Actions

Only 140 (2.1%) of all 6621 physician clinical-privileges reports during the study period were sexual-misconduct–related (Table 5). Overall, the proportions of sexual-misconduct–related clinical-privileges reports with revocations and with professional review employment or firing were greater than those of reports related to other offenses (29.3% vs 18.8%, *P* < .002; and 6.4% vs 1.5%, *P* < .001, respectively). Denial of clinical privileges was noted in a lower proportion of sexual-misconduct–related reports, compared with reports related to other offenses (2.9% vs 8.4%, *P =* .02). Involuntary resignations were noted in 1.4% of sexual-misconduct–related reports, compared with .3% of reports related to other offenses (*P* = .02).

**Table 5:** Adverse Clinical-Privileges Actions Taken Against Physicians in Sexual-Misconduct–Related vs Other-Offenses–Related Physician Clinical-Privileges Reports (Report-Level Analysis).

CP Actions^a^	Sexual-Misconduct–Related Reports (n = 140)^b^	Other-Offenses–Related Reports (n = 6481)^c^	*P* Value
	No. (%)	No. (%)	
Reports with revocation of clinical-privileges action	41 (29.3)	1218 (18.8)	.002
Reports with professionally reviewed firing action	9 (6.4)	97 (1.5)	< .001
Reports with voluntary surrender of clinical privileges under investigation action	40 (28.6)	1541 (23.8)	.19
Reports with involuntary resignation	2 (1.4)	20 (.3)	.02
Reports with denial of clinical-privileges action	4 (2.9)	541 (8.4)	.02
Reports with suspension of clinical-privileges action	28 (20.0)	1070 (16.5)	.27
Reports with summary/emergency suspension of clinical-privileges action	25 (17.9)	1061 (16.4)	.64
Reports with limitation/restriction of procedures/practice area action	2 (1.4)	86 (1.3)	.92
Reports with other unspecified restriction/limitation of clinical-privileges action(s)	12 (8.6)	734 (11.3)	.31

^a^ Each report can have up to five actions.

^b^ Reports are for 131 unique physicians with sexual-misconduct–related clinical-privileges reports.

^c^ Reports are for 5321 unique physicians with other-offenses–related clinical-privileges reports.

### Reported Type of Victim Injury in Malpractice-Payment Reports

“Emotional injury only” accounted for a significantly higher proportion of victim injuries in sexual-misconduct–related malpractice-payment reports than in reports related to other offenses (82.0% vs 1.5%, *P* < .001) (Table 3). In contrast, the proportions of victims reported to have died or sustained a type of major, significant, or permanent injury in sexual-misconduct–related reports were significantly lower than those in malpractice-payment reports related to other offenses.

### Inaction by State Medical Boards Concerning the Majority of Physician Sexual-Misconduct Reports From Other Sources

Physician-level analysis showed that of the 253 physicians with sexual-misconduct–related clinical-privileges or malpractice-payment reports, 177 (70.0%) had no sexual-misconduct–related licensure reports—ie, they were not disciplined for sexual misconduct by medical boards (Table 1). Of those 177 physicians, 92 (52.0%) had only clinical-privileges reports, 82 (46.3%) had only malpractice-payment reports, and three (1.7%) had both clinical-privileges and malpractice-payment reports related to sexual misconduct.

## Discussion

This is the first analysis of data involving physician sexual misconduct in the U.S. that includes adverse licensure and clinical-privileges restriction actions, as well as malpractice payments. Over the 10-year period of our study, only a small number of physicians (n = 1039) were reported to the NPDB due to sexual misconduct. Consistent with prior studies [13,21,22], we found that a disproportionate share of physicians with sexual-misconduct reports were aged 40 or older. More specifically, physicians aged 40 to 49 and 50 to 59 accounted for a significantly higher proportion of physicians with sexual-misconduct–related reports compared with the proportions these age groups represent in the general U.S. physician population.

Our study also offers the first national evidence concerning physician sexual-misconduct victims’ age, sex, and type of injury in malpractice cases that resulted in a payment being made on behalf of a physician. Overall, significantly more sexual-misconduct victims were female, and more than twice as many were 20 to 39 years old compared with victims in malpractice-payment reports related to other offenses. Our findings on physician sexual misconduct and victim characteristics can be informative to decision makers interested in focusing on these groups of physician offenders and at-risk victims.

Although it is expected that death and major injuries are unlikely to result from sexual misconduct, the fact that 82% of physician sexual-misconduct victims in malpractice-payment reports not only were able to pursue malpractice cases but also prevailed on the basis of “emotional injury only” suggests that the emotional injury was serious in these cases.

As supported by previous research [13], our licensure report analysis shows that when medical boards took disciplinary actions against physicians for sexual misconduct, their actions were more serious than actions for other offenses. Similarly, our analysis of clinical-privileges reports revealed higher proportions of revocations and firings in sexual-misconduct–related reports compared with reports related to other offenses.

Our finding that more than two-thirds of the physicians with sexual-misconduct–related clinical-privileges or malpractice-payment reports were not disciplined by any state medical board for such conduct is concerning, because the NPDB provides medical boards with access to all clinical-privileges and malpractice-payment reports for physicians with these reports in their respective states. This finding is consistent with previous NPDB-based research that showed that no medical board actions were taken against more than 50% of the physicians who had reports of serious clinical-privileges actions because of negligence or incompetence [23]. Therefore, there is room for medical boards to improve how they act on information related to physician misconduct, thus better meeting their obligation to protect the public.

The limitations inherent in the nature of our data should be considered. No information on the sex and specialty of physicians with sexual-misconduct–related reports is available in the NPDB’s public-use file. However, previous studies showed that physicians with sexual misconduct are predominantly males [21] and that psychiatrists typically account for a disproportionately high share of reported cases [12,13,22], although in recent years a growing number of cases have involved family physicians and obstetricians/gynecologists [24].

Another data limitation is that, of the three report types analyzed in this study, the NPDB documentation explicitly refers to patient victims only in malpractice-payment reports. We cannot state with certainty that in licensure and clinical-privileges reports the victims of sexual misconduct were patients (rather than professional colleagues or people outside the clinical-practice context). However, we believe, based on one author’s (REO) prior experience as an NPDB research executive for many years, that relatively few sexual-misconduct disciplinary actions taken by clinical peer-review committees involve victims who are not patients. Additionally, the 1998 U.S. study examining physicians with licensure actions due to sexual misconduct found that victims were clearly patients in 75% of cases [13].

We also note that there is not necessarily a one-victim-to-one-report relationship for licensure and clinical-privileges disciplinary reports. Complaints from multiple victims may result in a single disciplinary action.

By far, the main limitation of our study is that it only represents physician sexual misconduct that resulted in a licensure or clinical-privileges disciplinary action or a malpractice payment that was reported to the NPDB. As with other professional misconduct, not all physician sexual misconduct behavior results in a disciplinary action or a paid malpractice claim, and therefore not all such misconduct is captured in the NPDB. Understanding this context is critical to recognizing that our study inherently represents only the tip of the iceberg of physician sexual misconduct in the U.S. Specifically, patients who are victims of sexual misconduct are rarely willing to report sexual misconduct. Of patients who do file complaints against health care providers, “nothing happened” to the alleged perpetrator in 55 percent of cases [25]. Physicians are also unwilling, in the majority of cases, to report sexual misconduct by colleagues [26], and attorneys are typically reluctant to pursue malpractice claims with little prospect for substantial damage awards [27], such as sexual misconduct cases which usually tend to involve only emotional injury.

Moreover, significant variation, more than fivefold, exists among states in serious disciplinary action rates [28]. Thus, vastly differing effectiveness of medical boards in investigating and disciplining physicians, including those engaged in sexual misconduct, may be another reason for fewer sexual-misconduct–related disciplinary actions being reported to the NPDB.

These factors suggest that our findings may not be generalizable to all physician sexual misconduct in the U.S., because they do not include cases of physician sexual misconduct not reported to authorities or cases that did not result in a clinical-privileges disciplinary action lasting more than 30 days or a malpractice payment. Additionally, because of the corporate-shield loophole [29], our findings cannot be representative of physician sexual-misconduct malpractice payments that do not name the responsible physician. This has been suggested to lead to an underestimation in the number of paid malpractice claims by about 20 percent [30].

Finally, we note that the approach we have taken to compare physician sexual misconduct and related victim information with all other types of physician offenses is subject to limitations because of the wide heterogeneity of this “other” misconduct category.

Future research is needed to uncover the full extent of physician sexual misconduct in the U.S. In the absence of a large-scale random sample survey of patients, the most practical source of data might be complaints made to state licensing boards, in addition to actions taken by the boards.

## Conclusions

Our study informs policy makers, the medical community, consumers, and patient advocates about the extent and characteristics of physicians with sexual misconduct reported to the NPDB, the nature of sexual-misconduct–related disciplinary actions, and the characteristics of malpractice-payment victims. We found that two-thirds of physicians with either sexual-misconduct–related clinical-privileges actions or malpractice payments—both strong forms of evidence that sexual misconduct actually occurred—were not disciplined for sexual misconduct by medical boards. Therefore, we highlight the need to reform licensing-board discipline in sexual-misconduct cases. Furthermore, the entire medical community needs to increase efforts to prevent physician sexual misconduct and to be aggressive in reporting and disciplining physicians who engage in such misconduct. Particularly, more stringent state legislative oversight of medical boards is warranted to ensure that all physicians who have committed sexual misconduct face appropriate medical-board disciplinary actions for violating this most fundamental tenet of the physician-patient relationship.

## Supporting Information

S1 File Supplemental Methods. (DOCX)
