# Supplementary material for: Cross-Sectional Analysis of the 1039 U.S. Physicians Reported to the National Practitioner Data Bank for Sexual Misconduct, 2003–2013
Source: PLoS One. 2016 Feb 3;11(2):e0147800. doi: 10.1371/journal.pone.0147800 (PMC4739584; doi:10.1371/journal.pone.0147800)
Supplement: S1 File — (DOCX) [file pone.0147800.s001.docx]

AbuDagga et al.

Cross-Sectional Analysis of 1039 U.S. Physicians Reported to the National Practitioner Data Bank for Sexual Misconduct, 2003**–**2013

Supplemental Methods

*Identification of report types*

To identify SL and CP reports, we used report codes “301 = SL action legacy report” or “302 = SL action updated report,” and “401 = CP action legacy report” or “402 = CP action updated report,” respectively. MP reports were identified using codes “101 = insurance company MP” or “102 = non-insurance company MP.” SM-related reports were identified by code “D1 = sexual misconduct” in any of the five basis-for-action variables in SL or CP reports, or code “717 = sexual misconduct” in either of the two specific malpractice act or omission variables in MP reports.

*Age groups for the general U.S. physician population*

We obtained general physician population age counts from the Federation of State Medical Boards (FSMB) [1]. These counts were for 2010, as we were unable to identify publicly available counts for 2008.

**References**

1. Young A, Chaudhry HJ, Rhyne J, Dugan M (2011) A census of actively licensed physicians in the United States, 2010. J Med Regul 96:10–20.
